# Utilizing categorical boosting and SHAP to understand key predictors of frequency of use at discharge for completed substance abuse treatments in TEDS-D

**DOI:** 10.1088/3049-477X/ae9bc7

**Published:** 2026-10-07

**Authors:** C Flynn, S Abad, B Wooton, C Jansen, B Johnson, B Harrison, J Nash, A J Stokes, H Turner

**Affiliations:** 1 Laboratory of Pharmacology and Analytics, School of Natural Sciences and Mathematics, Chaminade University, Honolulu, Hawai’i, United States of America; 2 Data Analytics Research Team, UN CIFAL Honolulu, Chaminade University, Honolulu, Hawai’i, United States of America; 3 Data Science Program, School of Natural Sciences and Mathematics, Chaminade University, Honolulu, Hawai’i, United States of America; 4 NSF INCLUDES ALL-SPICE Alliance program, Chaminade University, Honolulu, Hawai’i, United States of America; 5 ScHARe Fellows Program, NIH AIM–AHEAD Consortium, Fort Worth, Texas, United States of America; 6 J. Craig Venter Institute, La Jolla, California, United States of America; 7 Habilitat, Inc., Kaneohe, Hawai’i, United States of America; 8 AIM-AHEAD AI Resource Concierge for Healthcare (ARCH) program, John A. Burns School of Medicine, University of Hawai’i, Honolulu, Hawai’i, United States of America; 9 Department of Cell and Molecular Biology, John A. Burns School of Medicine, University of Hawai’i, Honolulu, Hawai’i, United States of America

**Keywords:** substance abuse, TEDS-D, treatment outcomes, AI/ML, healthcare analytics

## Abstract

*Background:* Substance use disorder is a pressing US public health crisis, with 48.7 million people reporting past-year substance use and over 100 000 overdose deaths in 2022. Building on a growing body of machine learning research using the SAMHSA treatment episode Data set—discharges (TEDS-D), this study introduces *frequency of use at discharge* as an outcome variable and the inclusion of *U.S. state* as a predictor. *Research Design and Methods:* First, *reason for discharge* was evaluated as a dependent variable and compared to *frequency of use at discharge*. Utilizing 2411 351 completed treatment episodes from TEDS-D 2015–2019, a categorical boosting model was employed to predict *frequency of substance use at discharge* using 62 predictor variables. Shapley additive explanations (SHAP) calculated global feature importance and decomposed individual predictions. State-level models were compared to the national model using rank-biased overlap (RBO), with a Hawai’i case study demonstrating state-level decision support. *Results:* Although treatment completion yielded higher rates of *no use* at discharge than dropout (45% vs 28%), 25% of completed episodes reported active substance use, 9% reported homelessness, and 50% reported unemployment at discharge. The model demonstrated strong predictive performance (AUC = 0.96, sensitivity = 0.85, specificity = 0.95). Treatment and geographic factors outweighed patient attributes, with *frequency of use at admission, U.S state of treatment, length of stay*, and *service type* as the four most important features. Among all service types, long-term residential rehabilitation was associated with the lowest SHAP values and predicted probability of substance use at discharge. State-stratified models showed moderate feature importance consistency (mean RBO = 0.59, range: 0.30–0.73). The Hawai’i case study identified elevated probability of use at discharge for younger clients and revealed substantial underreporting of long-term residential and therapeutic community treatment modalities in federal data. *Discussion and Implications:* State-level variability in treatment outcomes reflects systemic inconsistencies in policy, reporting, and service access that complicate national benchmarking and may obscure the true drivers of patient recovery. Explainable ML provides actionable insights for tailoring treatment and informing data-driven policy to improve outcomes.

## 1. Introduction

The 2020 National Survey on Drug Use and Health (NSDUH) [1, 2] reported that 58.7% of Americans, equivalent to 162.5 million individuals, were current users of tobacco, alcohol, or illicit drugs. Additionally, 14.5% (or 40.3 million people) were diagnosed with a substance use disorder (SUD) [3, 4]. Among those aged 12 and older, 1.4% (or 4.0 million individuals) received any form of SUD treatment within the past year, and 1.0% (or 2.7 million people) sought treatment at a specialized facility [5]. It is well established that effective treatment can significantly reduce substance dependence and positively impact related areas, such as mental health, criminal justice involvement, and employment opportunities [6–8]. However, even those who complete treatment may continue to experience significant hardships. For instance, in substance abuse and mental health services administration (SAMHSA) treatment episode dataset—discharges (TEDS-D) [9, 10], 26% of individuals who complete substance abuse treatments are discharged as active users, 55% are either unemployed or not participating in the labor force, 18% live in dependent housing situations, and 10% are homeless. Understanding the factors that define success and contribute to the successful completion of treatment for substance abuse disorder is a research priority to inform decision support for both patient welfare and resource allocation [11–13].

Current frameworks for predicting treatment success in SUD rely on ingesting a range of factors (e.g. demographic characteristics, treatment modalities, psychosocial variables, and biological or clinical markers) into traditional statistical models such as logistic regression or survival analysis [14, 15]. They focus on identifying the predictors of whether an individual will successfully complete a treatment program or remain abstinent post-treatment using a strength of associations and statistical significance metrics. More broadly, the field recognizes that behavioral and psychosocial models and biopsychosocial models are strong predictive frameworks [16, 17] that incorporate a wide range of observed, self-reported and measure variables such as motivation to change, social support networks, psychiatric comorbidities, cultural context and readiness for treatment [18, 19]. However, these key variables are often self-reported, not captured in large Federal datasets or may lack the statistical weight as individual variables to appear as compelling predictive features, limiting their apparent penetrance into predictive models. Moreover, the reliance on static variables, such as demographics or single-event measures (e.g. discharge status), may fail to account for the dynamic nature of recovery and relapse risk [20]. Individuals’ needs, challenges, and resources also change over time, necessitating a more flexible approach to prediction. ML is particularly useful for identifying nonlinear relationships, interactions between predictors, and combined effects of weak predictors [21, 22]. Numerous Federal and other public datasets present numerous patient-intrinsic or -extrinsic (e.g. patient demographics and personal history versus location or type of treatment program) variables associated with treatment outcomes, allowing for use of both conventional statistical and machine learning approaches to associate these variables with treatment outcomes. However, the multifaceted interactions between variables and the disconnect between the milestone of treatment completion versus full recovery continue to complicate analyses and require innovation in ML approaches.

TEDS-D is a national dataset that provides detailed information on substance abuse treatment episodes in facilities across the U.S [9, 10]. Collected by the SAMHSA, it encompasses a wide range of demographic, treatment-related, and outcome variables [9, 10]. Given the complexity of substance abuse and its treatment, the TEDS-D dataset is well-suited for application of ML to detect patterns, relationships and interactions that are not immediately evident in conventional statistical approaches. Baird *et al* [23] examined racial disparities in TEDS-D outcomes using a two-stage ML method combining random forest and logistic regression. Automated ML has been used in TEDS-A (Admissions) to investigate interactions between variables [24, 25]. For example, interactions between mental health and SUD were converted into a strong predictive framework by combining predictions of random forest model and XGBoost models into a superlearning model [15, 26]. Completion of addiction treatment has been identified as one of the most consistent factors associated with a favorable treatment outcome [27]. Individual attributes such as age, education, employment, living arrangement, insurance, and race, have been shown to be associated with completing treatment [28, 29]. However, at least one predictive modeling study on opioid treatment has found that system-level factors, including treatment service setting, geographical region, primary source of payment, and treatment referral source, can be the strongest predictors of opioid treatment discontinuation, opposed to individual-level factors [30].

In this study we used 2411 351 million completed treatment episodes from 2015–2019 in TEDS-D and applied a categorical boosting model to rank patient-intrinsic and -extrinsic importance features associated with treatment success. We assessed the implications of using ‘reason for discharge’ (i.e. treatment completion or self-discharge) as a dependent variable, and comparatively examined completed versus dropped out treatment trajectories using four pre- and post-treatment data fields that are collected in TEDS-D, noting stagnancy in features that describe SAMSHA’s four dimensions of recovery: health, home, purpose, and community (HHPC) [31–33]. We evaluated the feature importance rankings assessing treatment outcomes and classified patient-intrinsic and extrinsic factors. We examined the underlying drivers of the primacy of geographical location (by state) as a feature associated with treatment outcomes, generating both insights into state-by-state variations in treatment modality and reporting variables. We assessed relationships between outcomes and a state’s position in a classification heuristic reflecting sociocultural, political and economic attitudes to SUD and then generated partial dependence plots comparing features other than geography by state. Finally, we examined Hawai’i as a case study in the strengths and limitations of TEDS-D analyses using these methodologies.

## 2. Materials and methods

### 2.1. Data source

TEDS-D [9, 10] is a standardized, national administrative dataset maintained by the SAMHSA within the U.S. Department of health and human services. It is designed to capture structured discharge-level data on individuals aged 12 and older who receive treatment for SUDs in publicly funded or state-licensed/certified specialty treatment facilities. TEDS-D includes treatment episodes from structured outpatient, inpatient, and residential settings. TEDS-D is part of the drug and alcohol services information system and is governed by state-level reporting requirements to single state agencies (SSAs) [34]. It excludes office-based private treatment settings unless state regulations mandate their inclusion.

### 2.2. Data and code availability

This study used the TEDS-D annual public-use files (PUFs) made available through the substance abuse and mental health data archive [35], with documentation including codebooks, import-ready formats (SAS, Stata, R), and user guides. All code can be found on GitHub (https://github.com/NSF-ALL-SPICE-Alliance/SAMHSA-treatment-ML) for review and reproducibility.

### 2.3. Data quality and security

The technical architecture of TEDS-D is based on a centralized relational database system managed through secure federal contractor infrastructure. States transmit data to SAMHSA using encrypted, secure file transfer protocols or web-based portals. Incoming files undergo automated validation routines that check for structural compliance, range violations, internal consistency, and adherence to format specifications defined in the TEDS crosswalk and data submission specifications manual. Rejected records are flagged and returned for correction prior to ingestion into the national dataset. TEDS-D metadata includes variable definitions, permissible values, missingness indicators, source coding protocols, and timestamped submission logs. Metadata and data dictionaries are maintained by SAMHSA and updated periodically as part of the TEDS technical assistance effort. Cyberinfrastructure supporting TEDS-D includes a secure data ingestion pipeline, centralized cloud-based storage and database management systems, and access-controlled analytic environments for approved users.

### 2.4. Data ethics

PUFs are de-identified in accordance with HIPAA and federal statistical confidentiality standards, with additional suppression applied to small cell sizes to protect privacy. TEDS-D is not classified as human subjects research under federal regulations and does not require institutional review board oversight for secondary analysis.

### 2.5. Data set inclusion criteria

TEDS-D records after 2015 contain improved granularity on patient characteristics and completeness. Although the most recent available data describes treatment episodes in 2022, this analysis focuses on the years 2015–2019 to avoid confounding effects related to the COVID-19 pandemic on substance abuse treatment and reporting (see Stafford *et al*) [30]. TEDS-D contains > 3.3 M completed treatment discharges from 2015 to 2019 alone.

### 2.6. Data filtering

Data filtering is described in detail in figure 1. Each TEDS-D record captures ∼76 standardized data elements, including variables related to treatment characteristics (e.g. modality, service setting, length of stay (LOS)), client demographics (e.g. age, gender, race/ethnicity, education, employment), substance use patterns (e.g. primary/secondary substances, frequency of use), and discharge outcomes (e.g. completion, transfer, administrative discharge). Data definitions and value codes follow national standards to enable longitudinal and cross-jurisdictional analysis, although some optional fields may vary by state. Data were filtered for only completed treatment episodes to exclude the individual subjectivity of patients dropping out. In order to perform binary classification, episodes completed with *some* or *daily* use at discharge were aggregated into one class. We note that 943 507 of completed treatment episodes did not report the patient’s frequency of use at discharge, therefore these episodes were dropped from the analysis. Significantly high variance in missingness of frequency of use at discharge is observed at the state-level with New Jersey (94% NA (not reported)), Maryland (91% NA), Arizona (69% NA), New York (60%NA), Nebraska (49% NA). This high missingness is not uniform nation-wide, with several states reporting ‘not reported’ or unknown frequency of use at discharge for < 5% of treatment episodes. The missingness in the discharge use frequency variable appears to follow a partially missing at random pattern [36]. It is strongly associated with observed factors such as the reporting state and patient frequency of use at admission but since there is elevated missingness among patients with high-frequency use at admission, missingness may also relate to unobserved discharge outcomes (potentially missing not at random, MNAR) [37]. Eleven features in TEDS-D with greater than 50% missingness were dropped from analysis. Final data included in the analysis is comprised of 2411 351 completed treatment episodes with reporting of frequency of use at discharge and 62 descriptive features.

**Figure 1.**
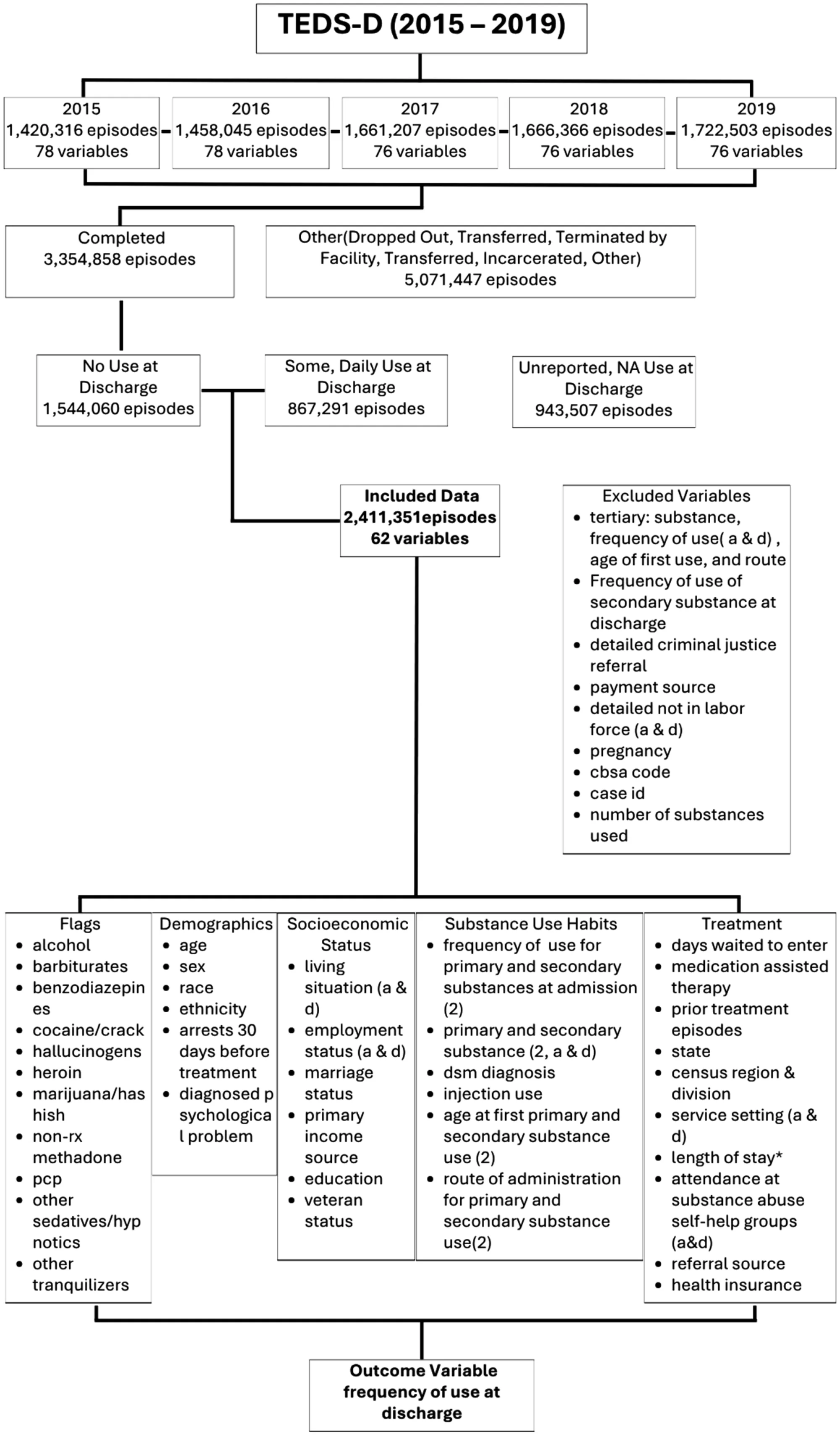
Data processing pipeline prior to initiation of machine learning. *Included* data indicates all data and features that were utilized in the machine learning model. NB: a & d indicates admission and discharge, such that the variable is reported twice.

### 2.7. Model selection, parameter tuning, and accuracy metrics

Data preparation and transformation were performed in R (v4.5.1) using *tidyverse* for data wrangling and *CatBoost* for model fitting. A categorical boosting model was utilized due to the categorical nature of the features in TEDS-D. CatBoost is a gradient boosting algorithm [26, 38] that successfully handles categorical features and takes advantage of dealing with them during training as opposed to preprocessing time. CatBoost uses a new schema, compared to previous gradient boosting techniques, for calculating leaf values when selecting the tree structure, which helps to reduce overfitting [38]. CatBoost works especially well when dealing with high-cardinality features which we see in TEDS-D features including state fips code (50 classes), primary substance use at admission (19 classes), DSM diagnosis (19 classes), etc. When a feature has missing values, CatBoost by default treats the missingness itself as informative rather than discarding it. Because all predictors in this analysis were treated as categorical features, CatBoost’s default handling assigns missing values their own distinct category during encoding, allowing the model to learn whether the absence of a value is itself predictive of the outcome. This default behavior was retained without modification; consequently, no separate imputation step was performed, as missing values were not estimated or replaced but instead preserved as an explicit category consistent with CatBoost’s standard categorical-feature treatment. Data were randomly partitioned into training (80%) and test (20%) sets at the episode level using a fixed random seed to ensure reproducibility; given the large sample size, no stratification was deemed necessary to ensure a representative distribution across splits. The learning rate was set to 0.1 in place of CatBoost’s automatic default, which tends toward more conservative values, as the large sample size reduces the risk of overcorrection at each boosting iteration. The number of boosting iterations was reduced from the default of 1000–500 to balance predictive performance with computational efficiency, and tree depth was retained at the default of 6 to maintain moderate model complexity. The model was trained with a log loss function for binary classification, using area under the curve (AUC), sensitivity, and specificity as the primary evaluation metrics.

### 2.8. Model interpretability

We addressed the issue of limited interpretability posed by ML methods using the shapley additive explanation (SHAP) interpretable machine-learning framework [39]. SHAP leverages game theory concepts to compute feature importance in two steps: training a classification model using all features in the initial interaction and then computing SHAP values for each feature, subsequently ranking them to identify the most significant features for modeling the target problem [39, 40]. SHAP reflects the influence of features in each sample and also generates global interpretation [41].

### 2.9. Analytics and visualization

Figures presented in this study were generated using R *ggplot*, and *shap* libraries. SHAP was used to generate both global and local interpretability visualizations. For global explanations, a feature importance bar chart displays the mean absolute SHAP value for each feature, reflecting its average marginal contribution to model output across all observations. SHAP value boxplots were constructed for each feature, displaying the distribution of SHAP values across feature classes to illustrate how different values of a feature influence model predictions. SHAP dependence plots were used to examine the relationship between a feature’s classes and their corresponding SHAP values, with points colored by a second feature to reveal potential interaction effects. For local explanations, waterfall plots decompose individual predictions into additive SHAP components, identifying which features drove a specific prediction toward or away from each outcome class.

### 2.10. State-level models

While the global model was trained on the full, processed and cleaned TEDS-D dataset, each local model was independently trained on features and outcomes on a single state. Treatment data was stratified by state to ensure that each state was proportionally represented in both training and test sets. The test data was also partitioned by state and evaluated using both the global and the corresponding state-specific models to assess the AUC for each state. Model calibration was assessed through log loss, and differences in predictive accuracy between the global and state-level models were tested using DeLong’s test for correlated AUCs [42]. Ranked-biased overlap (RBO) is a metric that quantifies the similarity between ranked lists and has been used previously in machine learning to compare feature importance lists produced by different models or by different datasets. RBO for the list of top 10 feature importance rankings for the global model and local model were calculated for each state using the Python package *rbo.* RBO values range from 0 to 1, where 0 indicates no overlap between the ranked lists and 1 indicates identical rankings. Analyses were conducted in R (v4.5.1) and Python (v3.9) with the tidyverse, CatBoost, shap, and rbo libraries.

### 2.11. Key variable definitions

The primary outcome variable, frequency of substance use at discharge (FREQ1_D), captures the reported frequency of primary substance use in the 30 d prior to discharge, coded as no use, some use, or daily use. Frequency of substance use at admission (FREQ1) follows the same coding structure and served as a key predictor variable. The treatment service type at discharge (SERVICES_D) categorizes the setting in which the client was discharged, ranging from 24-hour inpatient detoxification and residential rehabilitation to various levels of ambulatory outpatient care. Attendance at substance use self-help groups in the 30 d prior to discharge (FREQ_ATND_SELF_HELP_D) captures participation frequency in programs such as alcoholics anonymous and narcotics anonymous, ranging from no attendance to 8–30 times in the past month.’

## 3. Results

### 3.1. Study population

The final analytic sample comprised 2411 351 completed treatment episodes after excluding records with missing discharge frequency of use data. The sample was predominantly male (68%) and White (70%), with adults aged 25–34 representing the largest age group (33%). Alcohol (42%) and heroin (19%) were the most commonly reported primary substances at admission, and 40% of episodes reported daily use at admission. The outcome variable was moderately imbalanced, with 64% of episodes classified as no use at discharge compared to 36% reporting some or daily use (table 1).

**Table 1.** Study population. This table displays class distributions for 8 features describing the final study population.

Characteristic	*N*	*N* = 2411 351^a^
Age at admission	2411 351	
12–14		14 055 (0.6%)
15–17		75 477 (3.1%)
18–20		79 727 (3.3%)
21–24		218 494 (9.1%)
25–29		405 600 (17%)
30–34		388 395 (16%)
35–39		313 337 (13%)
40–44		228 119 (9.5%)
45–49		222 899 (9.2%)
50–54		212 070 (8.8%)
55–64		222 461 (9.2%)
65+		30 717 (1.3%)
Sex	2410 748	
Male		1644 755 (68%)
Female		765 993 (32%)
Missing/unknown		603
Race/ethnicity	2339 552	
Alaska Native (Aleut, Eskimo, Indian)		8237 (0.4%)
American Indian (other than Alaska Native)		77 154 (3.3%)
Asian or Pacific Islander		1342 (<0.1%)
Black or African American		354 742 (15%)
White		1640 936 (70%)
Asian		16 410 (0.7%)
Other single race		171 425 (7.3%)
Two or more races		57 623 (2.5%)
Native Hawaiian or other Pacific Islander		11 683 (0.5%)
Missing/unknown		71 799
Primary substance of use at admission	2389 023	
None		19 382 (0.8%)
Alcohol		1001 819 (42%)
Cocaine/crack		123 419 (5.2%)
Marijuana/hashish		304 995 (13%)
Heroin		448 473 (19%)
Non-prescription methadone		3257 (0.1%)
Other opiates and synthetics		141 234 (5.9%)
PCP		6036 (0.3%)
Hallucinogens		2929 (0.1%)
Methamphetamine/speed		282 582 (12%)
Other amphetamines		12 693 (0.5%)
Other stimulants		2382 (<0.1%)
Benzodiazepines		24 729 (1.0%)
Other tranquilizers		231 (<0.1%)
Barbiturates		813 (<0.1%)
Other sedatives or hypnotics		2809 (0.1%)
Inhalants		1278 (<0.1%)
Over-the-counter medications		1080 (<0.1%)
Other drugs		8882 (0.4%)
Missing/unknown		22 328
Frequency of use at admission	2362 060	
No use		682 604 (29%)
Some use		738 714 (31%)
Daily use		940 742 (40%)
Missing/unknown		49 291
Frequency of use at discharge (Outcome)	2411351	
No use		1544 060 (64%)
Some or daily use		867 291 (36%)
Treatment service type at discharge	2,411,351	
Detox—hospital inpatient		24 963 (1.0%)
Detox—freestanding residential		584 140 (24%)
Rehab—hospital		11 346 (0.5%)
Rehab—short term residential		376 882 (16%)
Rehab—long term residential		232 943 (9.7%)
Ambulatory—intensive outpatient		240 785 (10.0%)
Ambulatory—non-intensive outpatient		912 632 (38%)
Ambulatory—detox		27 660 (1.1%)
Length of stay	2411 349	
1 d		181 706 (7.5%)
2–10 d		535 124 (22%)
11–21 d		213 707 (8.9%)
22–30 d		225 838 (9.4%)
31–45 d		156 086 (6.5%)
46–60 d		115 647 (4.8%)
61–90 d		224 059 (9.3%)
91–120 d		190 414 (7.9%)
121–180 d		219 802 (9.1%)
181–365 d		247 641 (10%)
>365 d		101 325 (4.2%)
Missing/unknown		2

^a^ n (%) for all categorical variables; Missing/Unknown includes −9 coded responses.

### 3.2. Evaluation of TEDS-D frequency of use at discharge as a dependent variable

We evaluated several potential dependent variables for subsequent ML modeling. The overall landscape of reason for discharge in TEDS-D (over all 8 M records) shows that reasons for discharge comprise *treatment completed* (42%), *dropped out of treatment* (26%), *transferred to another treatment program or facility* (20%), *terminated by facility* (6%), and <5% *incarcerated, death or other*. Clearly not all of these are clinically relevant to patient substance use status. Figure 1 shows our workflow for assessing *frequency of use at discharge* as an outcome variable in this study. Figures 2(A)–(D) supports completing treatment is one of the most consistent factors associated with a favorable treatment outcome [31]. The difference between completed vs dropped out treatments with ‘no use’ at discharge is significant (45% and 28% respectively), however, clear challenges still remain for those completing treatment (25% still using and 28% unknown). Treatment episodes with ‘daily use’ at admission have the highest proportion of ‘unknown use’ at discharge, suggesting that the number of treatments in which the patient is still using at discharge of a completed treatment may be higher than 25%. Other features in the dataset are likely to determine treatment pathways in frequency of use from admission to discharge, particularly those framed by the SAMHSA HHPC ‘four dimensions of recovery’ [9, 10]. We compared changes in frequency of use (health), living arrangements (home), employment status (purpose), and attendance at substance use self-help groups (community) between admission and discharge for completed treatments and treatments ending due to the patients dropping out.

**Figure 2.**
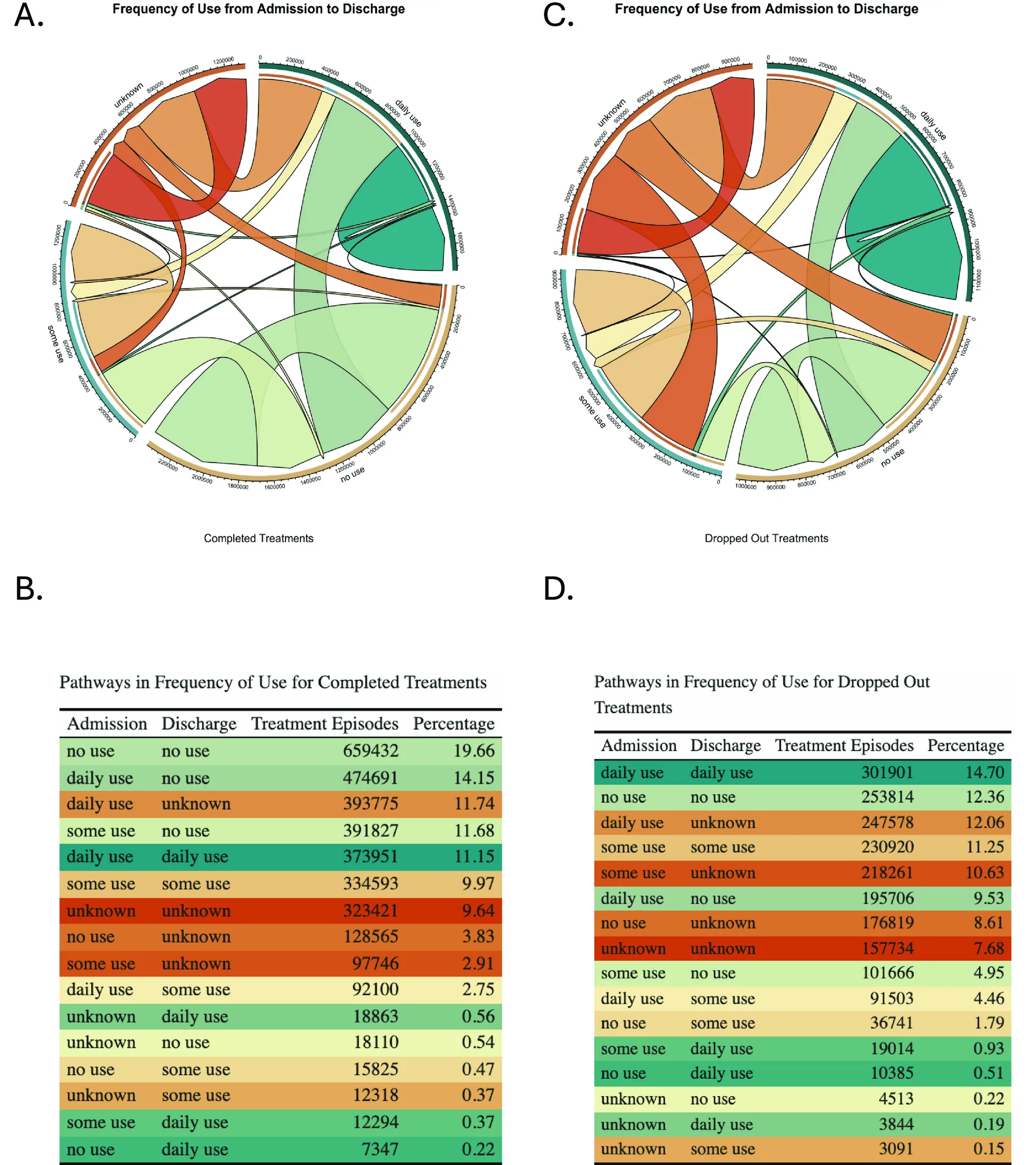
Transitions in treatment outcomes: chord diagrams and complementary tables illustrating changes in substance use, living arrangement, employment, and self-help group attendance from admission to discharge. (A) Chord diagram representing the change in frequency of substance use from admission to discharge for treatment episodes where the reason for discharge was ‘completed treatment.’ Flat lines indicate the frequency of use at admission, and arrowheads indicate the frequency at discharge. (B) Table complementary to figure 2(A), displaying all possible pathways in frequency of use from admission to discharge, along with the number and percentage of completed treatment episodes for each pathway. (C) Chord diagram representing the change in frequency of substance use from admission to discharge for treatment episodes where the reason for discharge was ‘dropped out of treatment.’ Flat lines indicate the frequency of use at admission, and arrowheads indicate the frequency at discharge. (D). Table complementary to figure 2(C). (E) Chord diagram representing the change in living arrangement from admission to discharge for treatment episodes where the reason for discharge was ‘completed treatment.’ Flat lines indicate the living arrangement at admission, and arrowheads indicate the living arrangement at discharge. (F) Table complementary to figure 2(E). (G) Chord diagram representing the change in living arrangement from admission to discharge for treatment episodes where the reason for discharge was ‘dropped out.’ Flat lines indicate the living arrangement at admission, and arrowheads indicate the living arrangement at discharge. (H). Table complementary to figure 2(G). (I) Chord diagram representing the change in employment from admission to discharge for treatment episodes where the reason for discharge was ‘completed treatment.’ Flat lines indicate employment at admission, and arrowheads indicate employment at discharge. (J) Table that is complementary to figure 2(I). (K) Chord diagram representing the change in employment from admission to discharge for treatment episodes where the reason for discharge was ‘dropped out.’ Flat lines indicate employment at admission, and arrowheads indicate employment at discharge. (L). Table that is complementary to figure 2(K). (M) Chord diagram representing the change in attendance at self-help groups from admission to discharge for treatment episodes where the reason for discharge was ‘completed treatment.’ Flat lines indicate in attendance at self-help groups at admission, and arrowheads indicate in attendance at self-help groups at discharge. (N) Table that is complementary to figure 2(M). (O) Chord diagram representing the change in attendance at self-help groups from admission to discharge for treatment episodes where the reason for discharge was ‘dropped out.’ Flat lines indicate attendance at self-help groups at admission, and arrowheads indicate attendance at self-help groups at discharge. (P) Table that is complementary to figure 2(O).

Figures 2(E)–(L) examines housing pathways and employment. These data demonstrate stagnancy in living arrangement from admission to discharge in both completed treatment and ‘dropped out’ treatments. The majority of patients stay in independent living arrangements from admission to discharge, regardless of whether they are completing treatment or not. We note that: (i) 307 639 treatment episodes indicate that patients completed treatment but remained homeless at discharge (9% of all completed treatment episodes), (ii)168 658 treatment episodes describe homeless patients that transitioned to dependent or independent living arrangements before completing treatment, (iii) 171 150 treatment episodes show homeless patients that dropped out of treatment and remained homeless (7% of all dropped-out treatment episodes), and (iv) 60 000 treatment episodes indicate patients that were homeless at admission transitioned to dependent or independent living arrangements, despite dropping out of treatment. Patients who are discharged as either unemployed or not in the labor force make up about 50% of episodes, regardless of reason for discharge. This is followed by patients keeping full time jobs through treatment (15% for patients who completed treatment and 9% for patients who dropped out. Improvement in employment status is scarce in completed treatment episodes with 4% of patients improving from not in the labor force at admission to unemployed at discharge, and 3% of patients improving from unemployed at admission to full time employment at discharge. Improvement in employment is slightly less (∼1% less) in treatment episodes ending with patients dropping out. Figures 2(M)–(P) displays changes in attendance at substance use self-help groups in the 30 d prior to admission and at discharge. Treatment episodes with no attendance or unknown attendance at discharge make up ∼56% of completed treatments and ∼70% of treatments in which the patient dropped out. About 11% of completed treatments episodes report patients attending self-help groups 8–30 times in the month prior to discharge, opposed to 4% for those dropping out. Notably, 173 328 completed treatment episodes (∼5%) demonstrated patients with no attendance at admission improving to attendance 8–30 times a month prior to discharge. Collectively these data on the SAMHSA ‘four dimensions’ and the selection of *frequency of use at discharge* as a dependent variable (figure 1) are foundations that set the stage for subsequent testing of the hypothesis that application of a categorical boosting model will identify other importance features linked to the HHPC framework.

### 3.3. Model accuracy assessment

The model correctly classified 293 292 instances where there was no substance use at discharge and 146 487 instances where some or daily use was reported. It misclassified 26 954 cases as no use when there was some or daily use, and 15 538 cases as some or daily use when there was none. These results demonstrate strong overall predictive performance, with a high true positive rate for both classes. The model achieved an AUC of 0.9627, indicating excellent discriminatory power in distinguishing between individuals who reported no substance use at discharge and those who reported some or daily use. This high AUC suggests that the model effectively captures underlying patterns in the data and can reliably predict post-treatment substance use status.

### 3.4. Aggregated feature importance in predicted substance use at discharge across national model

We next examined the aggregated feature importance values across all predictors in order to increase understanding of which factors most strongly influenced model predictions. Figure 3(A) displays a complete SHAP feature importance bar plot providing an untruncated view of the relative contributions of over 50 predictors used to model substance *frequency of use at discharge* from our national model. Treatment-related and geographic factors, such as *state, binned LOS*, and *service type at admission*, play a more significant role in predicting substance use outcomes than individual demographic and socioeconomic attributes, including race, education, income, veteran status, and *primary substance of use*.

**Figure 3.**
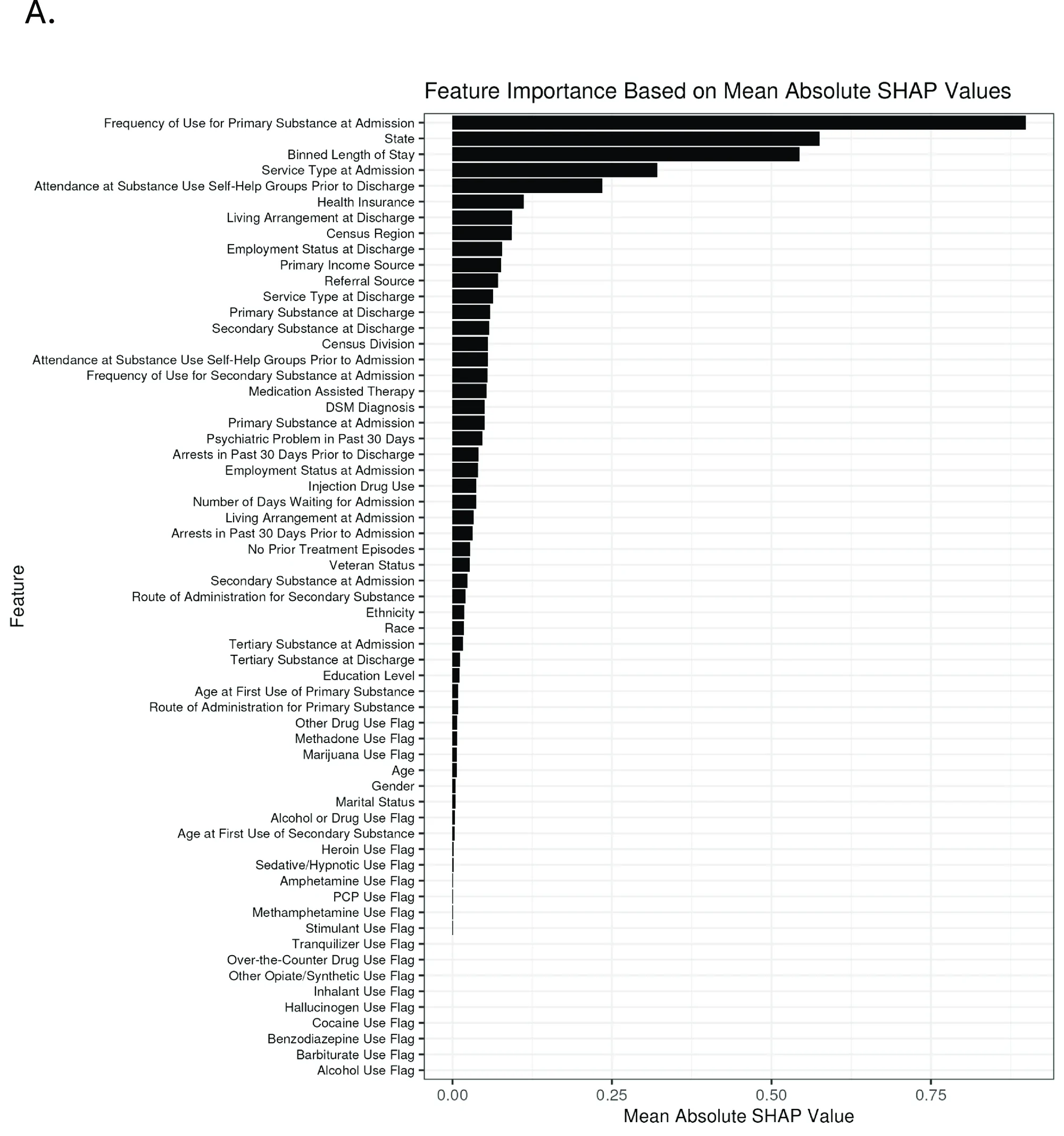
SHAP-based feature importance and dependence plots for predicting frequency of substance use at discharge using a CatBoost model. (A) SHAP feature importance plot based on mean absolute SHAP values from a CatBoost model predicting frequency of substance use at discharge. Each bar represents the average absolute SHAP value of a feature, indicating its overall contribution to model predictions. Features are ranked from most to least important. (B) SHAP dependence plot for the variable ‘frequency of use at admission.’ Each boxplot shows the distribution of SHAP values (i.e. the feature’s impact on model predictions) for each category of frequency of use at admission. Higher SHAP values indicate a stronger contribution toward predicting a higher likelihood of substance use at discharge, while lower (more negative) SHAP values indicate a contribution toward predicting a lower likelihood of use at discharge. (C) SHAP dependence plot for the variable ‘state.’ Each boxplot shows the distribution of SHAP values (i.e. the feature’s impact on model predictions) for each category of frequency of use at admission. Higher SHAP values indicate a stronger contribution toward predicting a higher likelihood of substance use at discharge, while lower (more negative) SHAP values indicate a contribution toward predicting a lower likelihood of use at discharge. (D) SHAP dependence plot for the variable ‘binned length of stay.’ Each boxplot shows the distribution of SHAP values (i.e. the feature’s impact on model predictions) for each category of frequency of use at admission. Higher SHAP values indicate a stronger contribution toward predicting a higher likelihood of substance use at discharge, while lower (more negative) SHAP values indicate a contribution toward predicting a lower likelihood of use at discharge. (E) SHAP dependence plot for the variable ‘service setting at admission.’ Each boxplot shows the distribution of SHAP values (i.e. the feature’s impact on model predictions) for each category of frequency of use at admission. Higher SHAP values indicate a stronger contribution toward predicting a higher likelihood of substance use at discharge, while lower (more negative) SHAP values indicate a contribution toward predicting a lower likelihood of use at discharge. (F) SHAP dependence plots for six categorical predictors of substance use frequency at discharge. Each panel shows the SHAP value distribution for different levels of a predictor variable, representing the feature’s influence on model predictions. Higher SHAP values indicate a stronger contribution toward predicting a higher likelihood of substance use at discharge, while lower (more negative) SHAP values indicate a contribution toward predicting a lower likelihood of use at discharge. Variables include attendance at self-help groups, health insurance type, living arrangement, census region, employment status, and primary source of income. Boxplots summarize the distribution of SHAP values for each category.

### 3.5. Explainable AI for important features and their individual classes impact on model prediction

The most important predictor we observe, *frequency of use for the primary substance at admission* (figure 3(B)) aligns with expectations based on the patterns observed in the raw data (figure 2(A)). Notably, the distinction between some use and daily use at admission appears relatively minor in determining *frequency of use at discharge*, while ‘no use’ at admission is of course a strong predictor of ‘no use’ at discharge. The second most influential variable in figure 3(C) is the state or territory of the US in which the treatment episode takes place. This is noteworthy due to the high cardinality (50 classes) of the feature. Features with large numbers of discrete categories often rank highly in importance scores but do not necessarily hold significant predictive power. However, figure 3(C) shows that the SHAP impact on prediction varies considerably by state. This suggests that systemic differences in state-level treatment approaches, reporting, policies, or funding may influence *frequency of use at discharge*, reinforcing the role of external structural factors in shaping treatment trajectories.

Figure 3(D) illustrates the influence of binned LOS on predicted frequency of use at discharge. The SHAP dependence plot shows a clear relationship between the duration of a treatment episode and frequency of use at discharge. Shorter stays, particularly one-day discharges and stays between two and ten days, are associated with higher SHAP values, indicating an increased likelihood of use at discharge. In contrast, longer treatment episodes tend to shift SHAP values closer to zero or negative, suggesting a reduced probability of substance use at discharge. This pattern aligns with established treatment research, where longer stays in structured treatment settings are generally associated with improved outcomes. However, the variability in SHAP values across different stay durations suggests that while LOS is an important predictor, it does not operate in isolation. Figure 3(E) demonstrates that long-term residential rehabilitation is associated with lower SHAP values, suggesting a decreased likelihood of substance use at discharge, while outpatient and short-term services show greater variability. The spread of SHAP values across service types highlights the heterogeneity in treatment experiences and outcomes, reinforcing that treatment setting alone does not fully determine frequency of use at discharge. We note that Therapeutic Community settings tend to be under-reported in TEDS-D [43, 44] (see Discussion) but these high-performing [45–47] long-term residential settings are likely to be associating with the lower SHAP value modalities.

Figure 3(F) describes the 5th–10th most important features in predicting frequency of use at discharge. Other than region, these features can be classified as patient intrinsic, providing insight into how patient characteristics affect model predictions of frequency of use at discharge. The SHAP dependence plots illustrate the impact of employment status, health insurance coverage, living arrangement, referral source, and self-help group attendance on model predictions. Employment status at discharge shows a distinction in SHAP values, with those classified as unemployed or not in the labor force exhibiting a higher likelihood of substance use at discharge. The distribution of SHAP values for health insurance status indicates that individuals with no insurance have a slightly higher predicted probability of use at discharge compared to those with Medicare, Medicaid, or private insurance. Living arrangement at discharge demonstrates a relationship with predicted outcomes, as individuals experiencing homelessness show higher SHAP values, while those in independent living situations have lower predicted probabilities of use. Referral source exhibits variability, with differences in SHAP distributions between court-mandated referrals, self-referrals, and other sources. Self-help group attendance in the past month is associated with differences in predicted discharge outcomes. The spread of SHAP values within each category suggests variability in individual responses to treatment, reinforcing the need to consider systemic and individual factors in understanding treatment episode outcomes.

Figure 4(A) assesses potential interaction effects by examining whether the SHAP values for treatment state vary systematically across levels of treatment duration, as measured by binned LOS. Longer treatment durations (greater than 21 d) are associated with lower SHAP values for treatment state, suggesting that the predictive influence of state on discharge outcomes diminishes with extended care. This trend is visualized through a general leftward gradient in color from yellow/orange (longer stays) to purple (shorter stays) along the SHAP value axis. However, the clarity and strength of this interaction vary by state. For example, Texas, Utah, and Pennsylvania exhibit a clear negative gradient, indicating a strong moderating effect of treatment duration on the impact of state. In contrast, Massachusetts, Mississippi, and North Carolina do not display consistent color trends, suggesting weaker or absent interaction effects between state and treatment duration in those settings.

**Figure 4.**
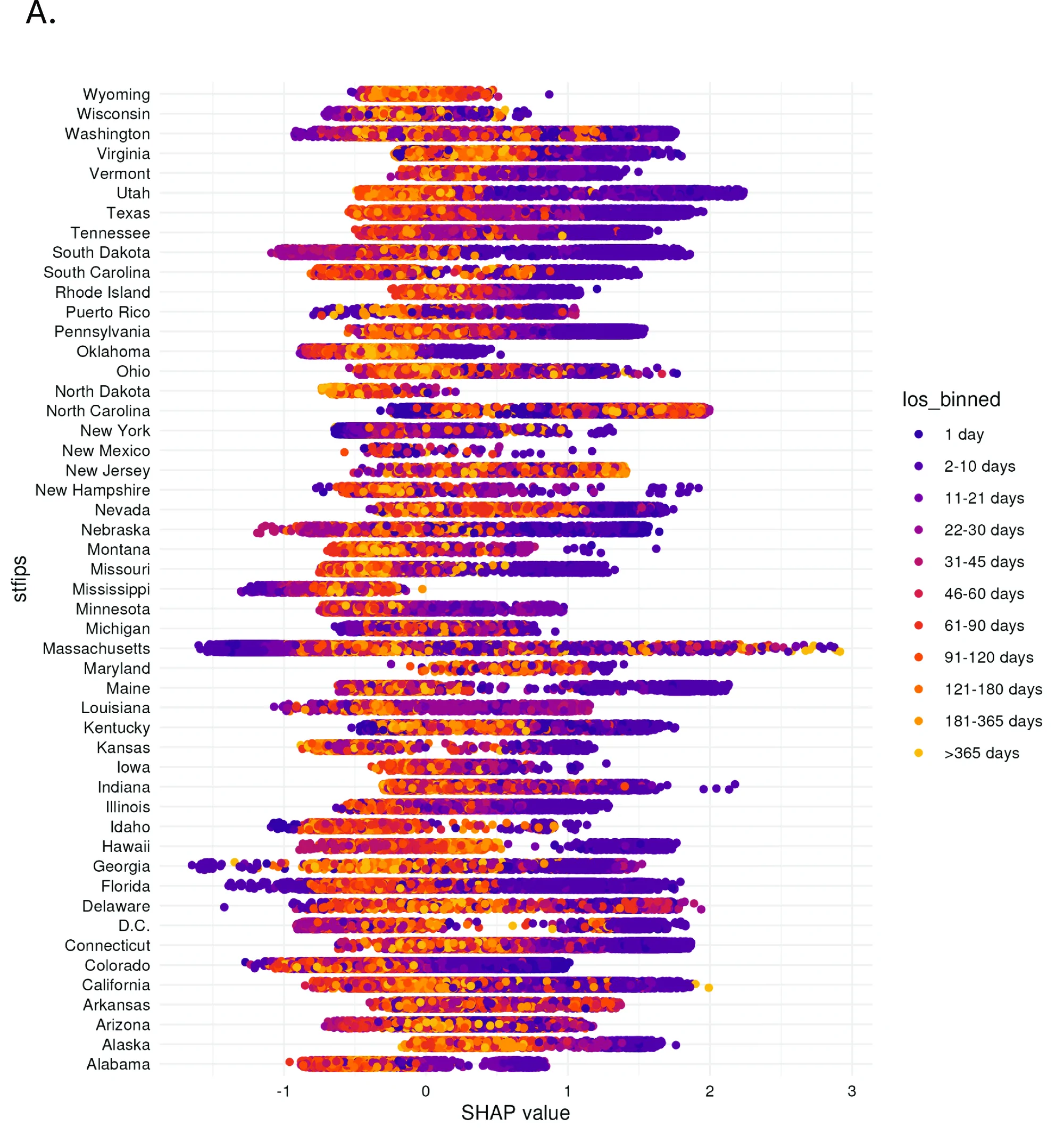
SHAP dependence plots showing the influence of U.S. state and interactions with treatment-related variables on likelihood of substance use at discharge. (A) SHAP dependence plot showing the main effect of U.S. state (FIPS code; stfips) on the predicted likelihood of substance use at discharge. Each point represents a treatment episode, with the horizontal axis indicating the SHAP value for stfips, reflecting its marginal contribution to the model’s prediction. Points are color-coded by binned length of stay (los_binned), allowing visual exploration of potential interaction effects between treatment duration and state. More positive SHAP values correspond to higher predicted probability of substance use at discharge, while more negative values indicate decreased predicted probability of substance use at discharge. (B) SHAP dependence plot showing the marginal effect of U.S. state (FIPS code; stfips) on the predicted likelihood of substance use at discharge. Each point represents a treatment episode, with horizontal position corresponding to the SHAP value for stfips, indicating its contribution to the model’s output. Points are color-coded by type of treatment service received at admission allowing for exploratory assessment of how service may condition or modify the effect of state. More positive SHAP values correspond to higher predicted probability of substance use at discharge, while more negative values indicate decreased predicted probability of substance use at discharge.

Figure 4(B) illustrates how the marginal effect of treatment state on the predicted probability of daily substance use at discharge is moderated by service setting at admission, relating to the data above (figure 3(E)) showing substantial variation in SHAP values across service types. Detoxification services were associated with consistently higher SHAP values, indicating that admissions to these settings tend to increase the model-predicted likelihood of use at discharge. In contrast, residential rehabilitation programs, particularly long-term residential care (more than 30 d), exhibit the lowest SHAP values, suggesting these settings are associated with a reduced predicted probability of use at discharge. This interaction pattern is re-investigated at the state-level in figure 4(B), where blue and teal points representing short and long-term residential services tend to cluster at lower SHAP values, while red and yellow points corresponding to detoxification services often appear at higher SHAP values. Intermediate variation is observed among orange service types, particularly ambulatory non-intensive and intensive outpatient care. The strength and clarity of this trend vary across states. For example, the interaction is clearly visible in states such as South Dakota and Hawai’i, more diffuse in Massachusetts and Georgia, and appears inverted in Louisiana, where residential services are associated with unexpectedly high SHAP values.

### 3.6. Considerations for interpreting the machine learning results connected to state

While SHAP values offer a robust framework for model interpretability, they are inherently tied to the structure and distribution of the training data. To contextualize the machine learning outputs, we present a series of exploratory visualizations of the raw feature distributions for the most influential predictors identified by the model. Figure 5(A) compares each U.S. state’s share of completed treatment episodes in the TEDS-D dataset (left, blue bars) with the state share of the total U.S. population (right, orange bars). The figure reveals substantial overrepresentation of certain states such as New York, Arizona, Massachusetts, Colorado, and Connecticut in the treatment data relative to their population share. Conversely, several other states including Texas and Florida are underrepresented in the TEDS-D data compared to their population size. As a result, the model’s learned relationships are disproportionately influenced by states with large volumes of treatment episode data, meaning that SHAP values for features such as treatment state, service setting, or demographics may reflect patterns that are more representative of these overrepresented states than of the national landscape. In contrast, states with relatively small TEDS-D sample sizes may produce unstable SHAP estimates or weak model signal, which can obscure meaningful relationships and limit the generalizability of feature effects in those regions. This is also an equity issue as small, and low resource states would be relatively underserved by decision support data. Figure 5(B) illustrates the variation in LOS with state level histograms colored by frequency of use at discharge. It is notable that the majority of completed treatment episodes reported by several states are less than or equal to 10 d including Arizona (50%), Colorado (70%), Delaware (62%), Florida (57%), Georgia (64%), Massachusetts (54%), Nebraska (50%), Nevada (60%), and New Mexico (51%). In most of these states, these treatments are completed with some or daily use reported at discharge, however, in Massachusetts almost all these short stays end with no use reported at discharge. New York, Arizona, and Rhode Island show patterns of reporting higher proportions of NA for frequency of use at discharge for stays ⩽ 10 d. This figure also shows the state level variation in the reported frequency of use at discharge with states including Idaho, Massachusetts, Montana, New Hampshire, Oklahoma, and Wyoming reporting no use at discharge for over 80% of reported, completed treatment episodes while Arizona, Delaware, Georgia, Maryland, Nevada, and New Jersey report <20% of completed episodes reporting no use at discharge. Much of this pattern can be explained by the state level variance in LOS, excluding Massachusetts, and state level variance in the proportion of treatment episodes in each service. Figure 5(C) illustrates state level variance in the proportion of treatment episodes in each service. At the national level, ambulatory non-intensive outpatient is the most utilized service at admission. States previously described with predominantly short LOSs including Colorado, Georgia and, Massachusetts report significant proportions of treatment episodes in the Detox, 24 h, freestanding residential setting at admission, 68%, 65%, and 75% respectively. Notably, Louisiana has the highest utilization of the short-term residential rehab setting (72% of treatment episodes) with a spike in figure 5(B) at 22–30 d LOS, however, those patients are predominately reported to be still using at discharge. States with higher utilization of residential short- and long-term settings, including tend to be highly ranked relative to SHAP values in figure 3(C) and demonstrate longer LOSs in figure 5(B).

**Figure 5.**
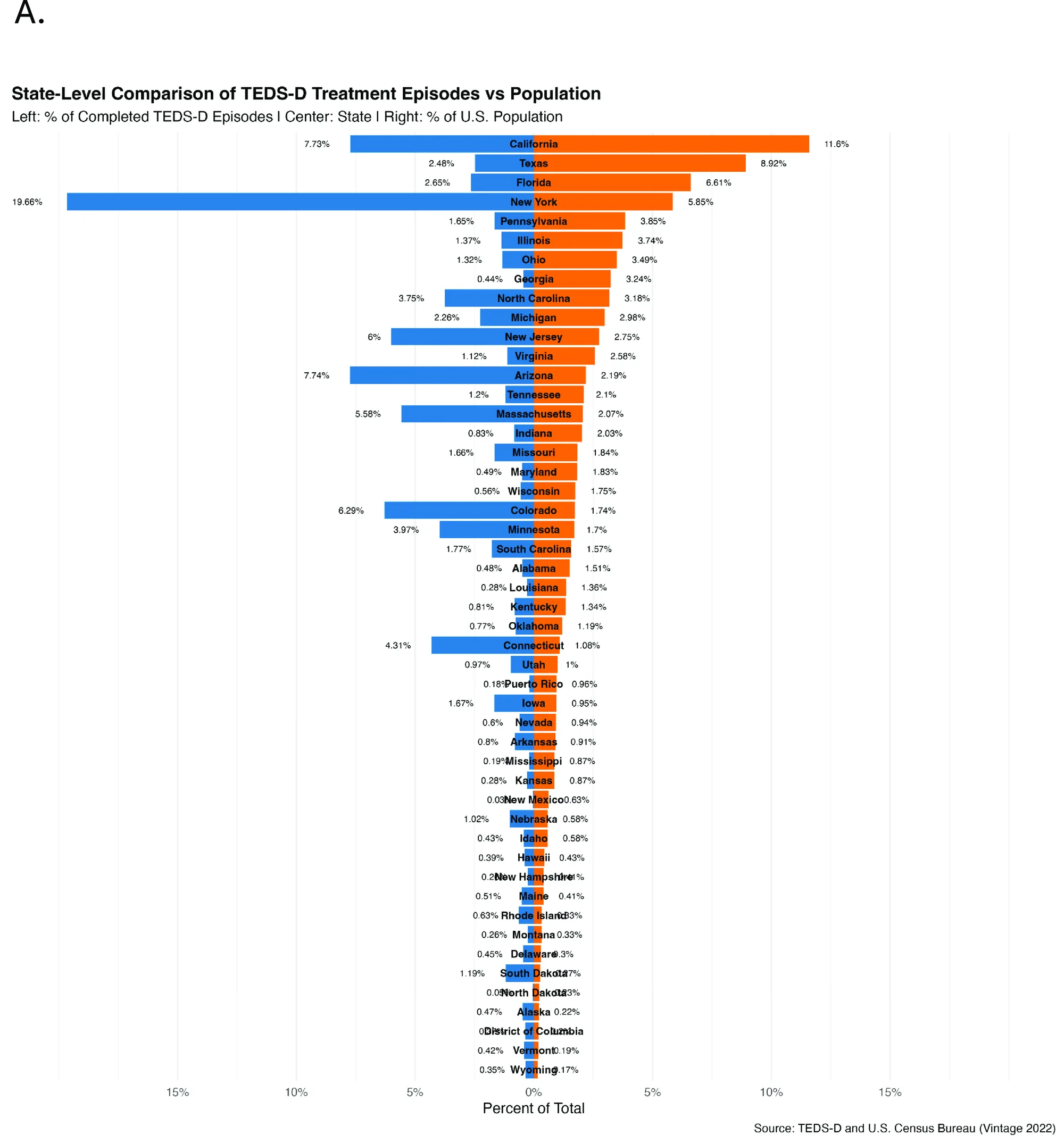
State-level comparisons of treatment representation, length of stay distributions, and service type patterns in completed treatment episodes. (A) Pyramid plot comparing each U.S. state’s proportion of treatment episodes in the treatment episode data set—discharges (TEDS-D; left, blue bars) to its proportion of the total U.S. population (right, orange bars). The mirrored layout allows for visual comparison of actual versus expected representation by state. States with longer blue bars than orange are overrepresented in TEDS-D relative to their population size, while those with longer orange bars are underrepresented. (B) Faceted histograms showing the distribution of binned LOS among completed treatment episodes in each U.S. state, colored by frequency of substance use at discharge. Each panel represents one state, with bars indicating the number of treatment episodes within each length-of-stay bin. Fill color corresponds to frequency of substance use at discharge categories, allowing joint visualization of treatment duration and post-treatment substance use outcomes. This layout highlights both within-state variation and cross-state differences in treatment length patterns and outcome distributions reported in the TEDS-D dataset. (C) Tile map displaying the distribution of completed treatment episodes by service type and state. Each tile represents the percentage of completed episodes in a given service category within a specific state. Color intensity reflects the relative proportion, with higher percentages shown in lighter yellow shades and lower percentages in darker purple shades.

### 3.7. Explainable AI for individual treatment episodes

Figures 6(A)–(D) present SHAP waterfall plots for four individual treatment episodes involving clients from Hawai’i, each age 20–29 with methamphetamine as the primary substance of use. These plots visualize how the model predictions for substance use at discharge are constructed additively from each feature’s SHAP value, with respect to the model baseline (*E*[*f*(*x*)] = 0). Each plot decomposes the prediction into additive feature effects, offering a granular view of the factors driving model output for a given treatment episode. Figure 6(A) demonstrates the model’s predicted probability of use at discharge is *f*(*x*) = 0.284. The largest positive contributor to the predicted probability was ‘some frequency of use at admission’, which increased the prediction by +0.643, reflecting the model’s learned association between prior use and continued use. In contrast, a long treatment duration (181–365 d) substantially reduces the prediction (−0.535), suggesting that extended care is associated with improved outcomes. Additional contributors include lack of self-help group attendance (+0.178), no health insurance (+0.095), and being treated in Hawai’i (+0.0894), while full-time employment at discharge (−0.132) and the other combined 52 features (−0.113) lower probability of use at discharge. While figure 5(A) depicted a moderate predicted probability of use at discharge (*f*(*x*) = 0.284), the model’s prediction in figure 6(B) is substantially lower (*f*(*x*) = − 1.14), indicating a much lower risk profile. As in figure 6(A), prior use at admission (‘some frequency of use’) increases the prediction (+0.419), though to a lesser extent than in the previous case. Notably, the most significant reduction in predicted risk comes from a very long treatment duration (more than 365 d, (−0.588). Full-time employment at discharge (−0.347) and the combined 52 other features (−0.319) contribute more strongly to risk reduction in this case. However, the client’s homelessness at discharge, despite an extended treatment episode and gaining full-time employment during treatment, adds risk (+0.156). Treatment in Hawai’i marginally lowers the predicted SHAP value contrary to figure 6(A) Figure 6(C) presents a predicted probability of use at discharge of *f*(*x*) = − 3.65, the lowest among the four treatment episodes. Unlike the prior cases, this client reported no substance use at admission, which is the largest negative contributor to the prediction (−1.31), consistent with the model’s strong reliance on baseline use behavior. A moderately long treatment duration of 91–121 d further decreases risk (−0.55), as does frequent attendance at self-help groups prior to discharge (−0.47). Several additional factors contribute negatively to the prediction, including placement in a long-term residential program at admission (−0.437) and at discharge, being treated in Hawai’i (−0.367), while medicaid health insurance is responsible for the only notable increase in SHAP value. Figure 6(D) presents the treatment episode with the highest predicted probability of use at discharge across the four cases, with *f*(*x*) = 6.58. In contrast to prior figures, the strongest positive contributor to the prediction is treatment in Hawai’i (+1.68), suggesting that, in this case, the model associates the client’s state of treatment with substantially elevated discharge risk. This is followed by a short LOS of 2–10 d (+1.13) and daily frequency of use at admission (+1.04), both of which reinforce the model’s pattern that high baseline use and minimal time in care are linked to poor outcomes. Other notable contributors include admission to a 24-hour, free-standing residential detox service (+0.74) and limited self-help group attendance prior to discharge (+0.572). Structural vulnerabilities such as lack of health insurance and homelessness at discharge also push the prediction upward. Minor positive contributions come from light self-help group attendance prior to admission and discharge from the same detox which together contribute less but remain directionally consistent with the overall risk profile.

**Figure 6.**
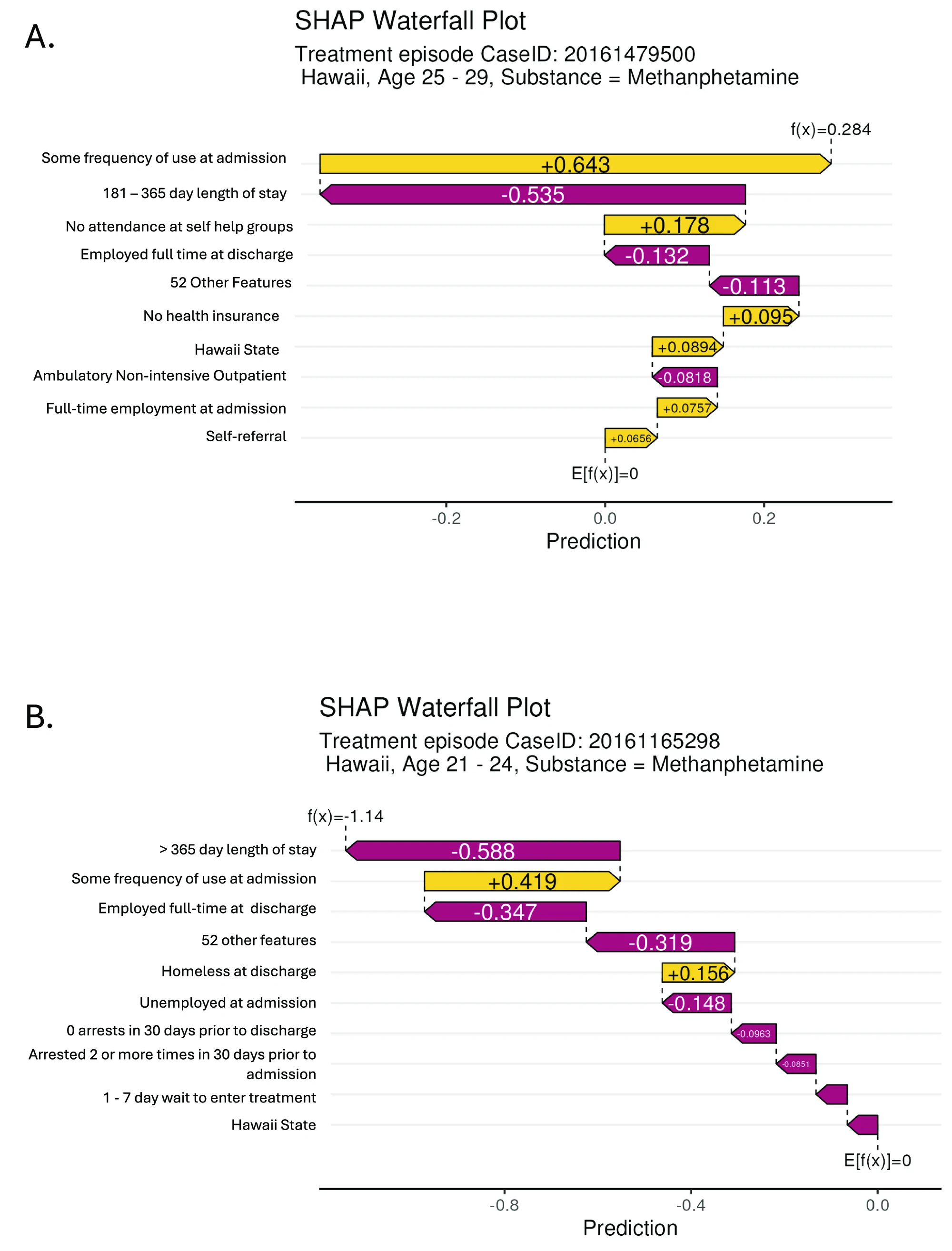
SHAP waterfall plots illustrating individual feature contributions to model predictions of substance use at discharge. (A)–(D) SHAP waterfall plot illustrating the contribution of individual features to the predicted likelihood of substance use at discharge for a single treatment episode. Bars represent the additive contribution of each feature to the model output, starting from the expected baseline prediction (*E*[*f*(*x*)] = 0). Yellow bars indicate positive contributions (increasing the predicted likelihood of substance use at discharge), while red bars indicate negative contributions (decreasing the predicted likelihood of substance use at discharge).

### 3.8. State level variation in feature importance

In addition to a global (i.e. nationwide) model, we deployed state-specific models to capture predictive features specific to each state. This approach was taken to evaluate whether tailoring models to state-specific data could better capture local patterns in predictive features, potentially improving predictive performance over a global model. Geographic variations in service access, service quality, policies may contribute to place-based differences in treatment outcomes across the regions which highlight the need for localized models to highlight predominant predictive features. Figure 7(A) demonstrates that for 46 of 49 states and territories, AUC improved under the local models. The three states with worse performance under a local model were states with more limited observations in the dataset (i.e. New Mexico, Montana and North Dakota) suggesting that limited datasets may benefit from a larger, more generalized dataset to avoid overfitting. To compare the similarity in predictive features between global and local models, we analyzed the consistency in feature importance rankings. Feature importance of the global model was compared to the feature importance of each local model using a measurement known as RBO. RBO is a metric that quantifies the similarity between ranked lists and has been used previously in machine learning to compare feature importance lists produced by different models or by different datasets. RBO for the list of top 10 feature importance rankings for the global model and local model were calculated for each state using the Python package *rbo.* Rbo values range from 0 to 1, where 0 indicates no overlap between the ranked lists and 1 indicates identical rankings. Figure 7(B) demonstrates RBO for each state, showing a significant range at the state level. Feature importance was calculated for each local, state-level model and the most importance feature in predicting frequency of use at discharge for each state is displayed in figure 7(C) Frequency of use for the primary substance at admission is the most important feature for prediction in most states, as would be expected. However, there are several exceptions. Service setting is the most important feature for Hawai’i, Alaska, Kansas, Colorado, Nebraska, Tennessee and Maine. The most important feature for California, Nevada, and Utah is the binned LOS. The number of days waiting for admission rises as the top predictor for Louisiana and Massachusetts. Attendance at self-help groups prior to discharge and referral source are the strongest predictors for Vermont and Mississippi respectively.

**Figure 7.**
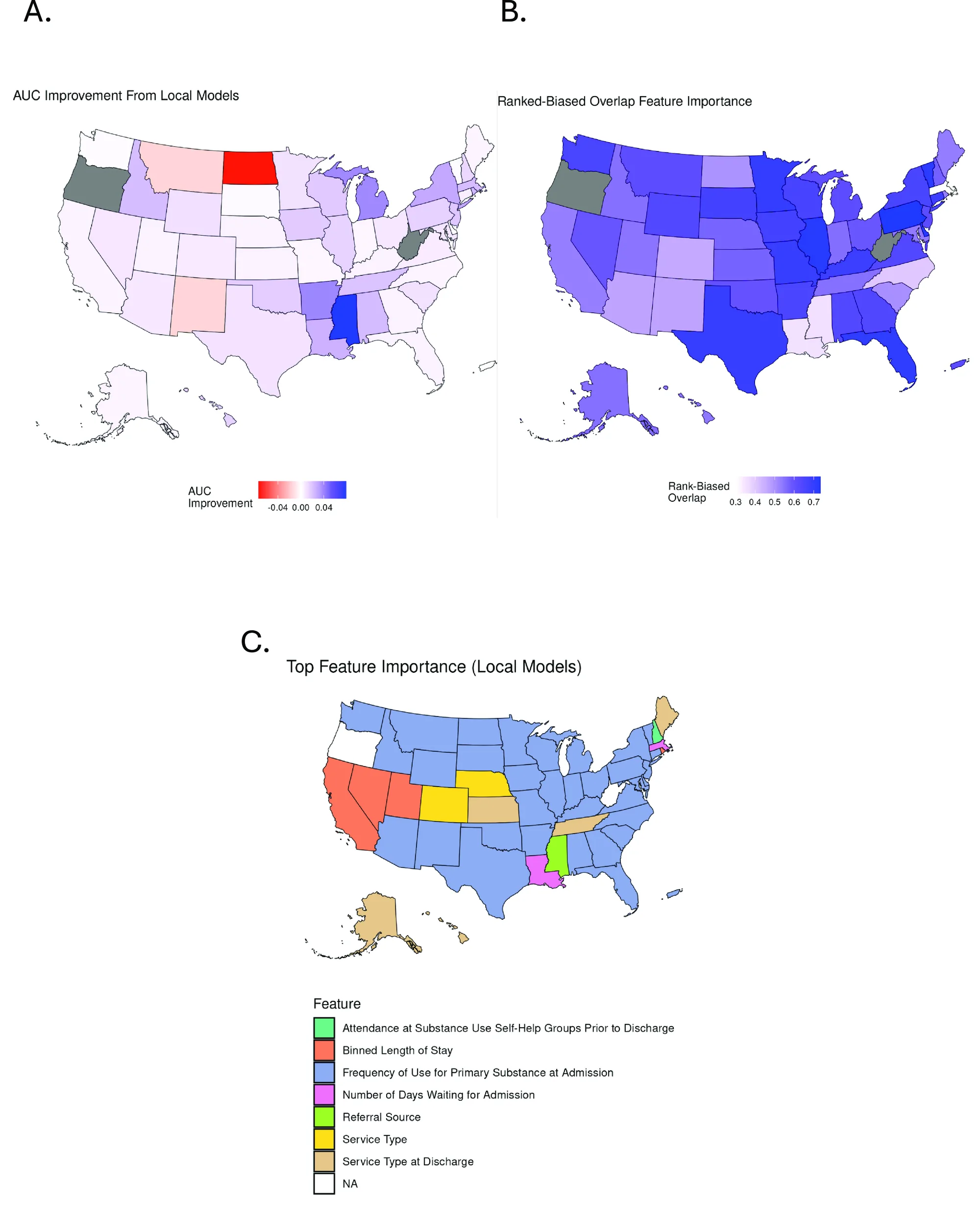
Geographic variation in model performance, feature importance alignment, and dominant predictors across U.S. states. (A) Choropleth map showing the change in model performance, measured as the difference in area under the curve (AUC), between state-specific models and a national model predicting substance use at discharge. Positive values (blue) indicate that a locally trained model outperformed the national model in that state, while negative values (red) indicate a decrease in AUC with local modeling. White indicates no appreciable change, and gray denotes states with insufficient data for evaluation. (B) Choropleth map showing rank-biased overlap (RBO) scores for feature importance rankings between each state-level model and the national model predicting substance use at discharge. Higher scores (darker shades of blue) indicate greater alignment in the ranked feature importance between the local and national models, while lower scores (lighter shades) reflect divergence in the most predictive features. RBO is a weighted similarity metric that emphasizes agreement at the top of the ranking lists. (C) Choropleth map showing the most important predictive feature in each state’s local model for predicting substance use at discharge. Each state is colored according to the feature with the highest importance score, indicating which factor contributed most to model predictions for that state’s treatment episodes.

### 3.9. A Hawai’i case study

We examined Hawai’i as a focused case study because geography emerged as a top predictor in our national models and state-specific models generally outperformed the global model, indicating meaningful local signal. Hawai’i’s treatment landscape is atypical because: (i) the service mix is skewed toward outpatient and medication-assisted care, (ii) long-term residential/therapeutic community capacity is under-captured in TEDS-D but is a cornerstone of culture-and community based treatment approaches in Hawai’i, and (iii) reporting patterns differ from state administrative sources (state alcohol and drug abuse division, ADAD) and facility surveys (national substance use and mental health services survey, N-SUMHSS). We hypothesized that these structural and reporting differences, combined with island geography and uneven service distribution across counties, may shape discharge outcomes in ways obscured by national aggregation. Initial finding showed that ML outputs for the Hawai’i -specific model largely mirror those observed in the national model but with some key differences. As shown in figure 8(A), the relative feature importance rankings are broadly consistent, with the exception of four variables (i.e. age, gender, frequency of secondary substance use at admission, and core based statistical area (CBSA) code which appear in the bottom half of the top ten most influential features in the Hawai’i model but not in the national model. Notably, CBSA was excluded from the national model due to high rates of missingness across states, but its inclusion in the Hawai’i model reflects more complete and localized reporting. Figure 8(B) presents SHAP dependence plots for the Hawai’i model and reinforces previously observed trends, including long-term residential rehabilitation and lengths of stay exceeding 21 d being associated with the lowest SHAP values, consistent with improved outcomes. However, several Hawai’i -specific patterns emerge. In particular, clients under the age of 24 exhibit elevated SHAP values, suggesting increased model-predicted risk of daily use at discharge among younger clients. Additionally, treatment in the Urban Honolulu CBSA is associated with higher SHAP values relative to those in Hilo-Kailua and Kahului-Wailuku-Lahaina, indicating potential geographic variation in treatment context, patient characteristics, or systemic factors influencing outcomes.

**Figure 8.**
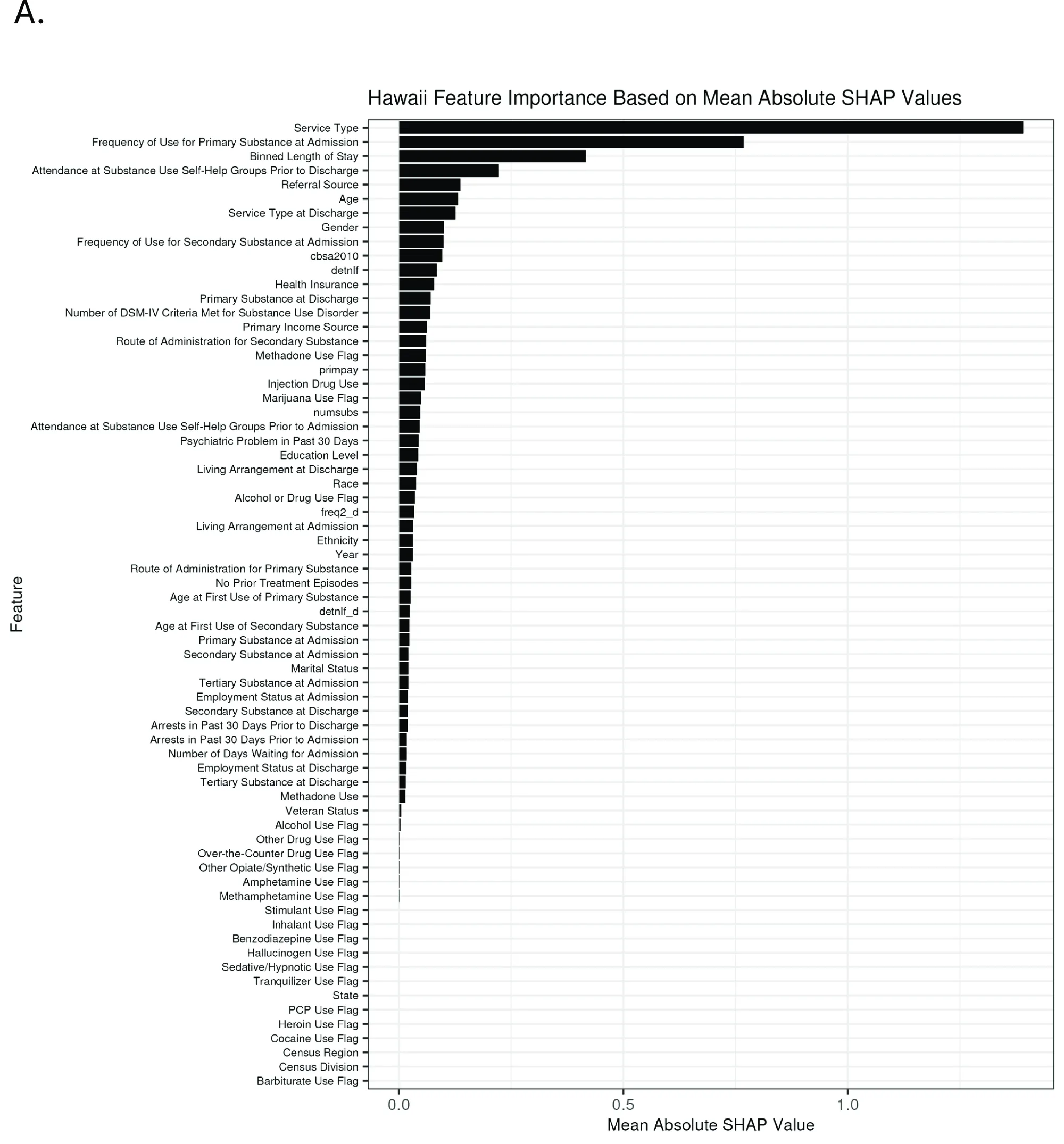
Hawai’i-specific machine learning analyses: SHAP feature importance, dependence plots, service combinations, and sequential treatment features. (A) SHAP feature importance plot utilizing only Hawaii data based on mean absolute SHAP values from a CatBoost model predicting frequency of substance use at discharge. Each bar represents the average absolute SHAP value of a feature, indicating its overall contribution to model predictions. Features are ranked from most to least important. (B) SHAP dependence plots utilizing only Hawaii data, for six key categorical predictors of substance use frequency at discharge. Each panel shows the SHAP value distribution for different levels of a predictor variable, representing the feature’s influence on model predictions. Higher SHAP values indicate a stronger contribution toward predicting a higher likelihood of substance use at discharge, while lower (more negative) SHAP values indicate a contribution toward predicting a lower likelihood of use at discharge. Variables include attendance at self-help groups, health insurance type, living arrangement, census region, employment status, and primary source of income. Boxplots summarize the distribution of SHAP values for each category. (C) UpSet plot showing the combinations of substance use treatment services offered by facilities in Hawai’i, based on data from the national substance use and mental health services survey (N-SUMHSS). Each vertical bar represents the number of facilities that offer a specific combination of services, with connected black dots below indicating which services are included. Bar colors denote the county in which the facilities are located. The left-hand bar chart shows how many facilities offer each individual service. (D) Sankey diagram visualizing 4 features reported at admission, binned LOS, service at discharge, and 4 features reported at discharge. This visualization shows the chronological nature of features reported in TEDS-D.

This case study also explores how completely substance abuse treatment activity in Hawai’i is reflected within the TEDS-D reporting framework. From 2015–2019 there were 43 000 substance abuse treatment episodes in Hawai’i reported to TEDS-D including 9500 in a long-term residential setting with 1500 completed (1800 dropped out, 5000 transferred, 1000 terminated by facility). Interestingly, differential reporting is occurring because for the same time period, the ADAD reported 3647 long term residential substance abuse clients. N-SUMHSS is an annual survey of US substance use and mental health treatment facilities States. Figure 8(C) displays services offered at the 30 facilities in Hawai’i documented in N-SUMHSS. Three facilities offer short term residential treatment, however, there are no short-term residential treatment episodes reported to TEDS-D in Hawai’i. Seven facilities offer long-term residential treatment, notably these facilities are only in Honolulu and Hawai’i counties. The majority of facilities offer outpatient treatments, and eight facilities offer methadone, buprenorphine, or naltrexone treatment., a significant bias towards medication-assisted treatment models.

## 4. Discussion

This study demonstrates that machine learning approaches can surface both expected and novel patterns in national substance use treatment data. Consistent with prior work, completion of treatment and lower baseline frequency of use at admission emerged as strong predictors of frequency of use at discharge [13]. The current findings extend beyond these individual-level factors, underscoring the centrality of structural and systemic elements (particularly state of treatment, service setting, and LOS) in shaping outcomes [12, 13]. The CatBoost framework, with SHAP-based interpretability, offered a transparent means of disentangling these interactions, and the data suggest that treatment context and geography (patient extrinsic factors) often outweighed patient demographics (patient intrinsic factors) in determining discharge trajectories. The decision to not exclude geography provided a useful contrast to practices in other TEDS-D studies that focus on ‘other than geography’ variables, or which include region-level variables but omit state of treatment due to the high cardinality of the variable potentially inflating importance scores [23, 32]. The deliberately minimal filtering applied to the dataset in this study allowed systemic features to emerge as key differentiators in determining whether an individual is reported using or not using their primary substance at discharge.

Several particularly interesting findings emerged in this study. First, the prominence of state as a predictor highlights that treatment outcomes are conditioned not only by clinical pathways but also by policy, funding, and reporting structures. The observed SHAP distributions suggest that ‘state’ is less a proxy for geography *per s*e than a bundled representation of systemic influences such as Medicaid coverage rules, residential bed availability, and block grant allocations. Second, the Hawai’i case study demonstrates how local modeling can both validate and complicate national patterns: while long-term residential treatment and extended LOS were again associated with improved outcomes, younger clients and those in the Urban Honolulu CBSA were flagged as higher-risk [48, 49]. These are patterns that would have been obscured in aggregated national models. Finally, the study suggests how under-reporting of therapeutic communities (TCs), which are widely regarded as effective and culturally resonant [50] models, creates measurement gaps that bias national analyses toward outpatient and medication-assisted treatments. These findings collectively argue for a more granular, context-sensitive approach to both modeling and policy interpretation. TC are high-performing community-centered treatment models [45–47] that are particularly successful in culturally- and community-grounded settings that work with psychosocially complex individuals [43]. TC form ∼10% of residential treatment beds in the US but < 8% of Federal TEDS-D [44] discharge data are from residential settings, which is also an aggregated category containing both TC and non-TC settings [28]. This TC underrepresentation in national SUD treatment datasets limits: (1) empirical effectiveness assessment and continuous improvement of TC, (2) informed resource allocation and policymaking, and (3) deployment of AI/ML models that can provide decision support for TC. Hawai’i is particularly impacted by TC data gaps. TC are relatively common in the state, and closely aligned with cultural and community values [51, 52], but struggle to affirm their effectiveness and make the case for support because of their limited footprint in Federal data. TEDS-D under-reporting of TC arises from three factors [9, 10, 46]: (1) TCs are less likely to receive state or federal funding, making them less likely to report to SAMHSA-mandated systems; (2) TC programs often operate independently or as part of nonprofit networks with limited technical capacity for standardized reporting; and (3) the structure of TEDS-D itself, which favors fixed-length, single-episode care models, is less suited to the long-term, multi-phase continuum of TC recovery. This structural opacity can obscure the effectiveness of TC models and prevent them from being fully included in data-driven policy or funding decisions. We suggest that the future closing of this data gap requires authentic collaboration between AI/ML practitioners and knowledge holders in the SUD TC with a next phase of research mapping patient trajectories using ‘small data’ to generate the type of trajectory analysis exemplified in figure 8(D).

The study has limitations that affect the interpretation of results and suggest avenues for future work. Machine learning models capture association rather than causality; selection into services and lengths of stay is non-random and may additionally reflect unobserved factors such as severity, motivation, or local resource constraints. Missingness in the discharge use variable is both state-dependent and plausibly not at random (MNAR), particularly for those with high baseline use, raising the risk of inflated abstinence estimates in some jurisdictions [53, 54]. The high cardinality of categorical features such as state can also produce inflated importance scores, even when predictive power is uneven. Moreover, TEDS-D systematically under-captures TCs and other long-term modalities, leading to structural underestimation of their effectiveness. For smaller or lower-resource states, limited episode counts constrain the stability of state-specific models, underscoring equity challenges in deriving decision support from nationally pooled data. Together, these shortcomings highlight the need for caution in translating SHAP-based insights directly into prescriptive policy without triangulation from complementary data sources.

The Hawai’i case study deserves particular attention because it illustrates both the strengths and blind spots of national reporting systems. Hawai’i’s local model mirrored national results by confirming the importance of treatment duration and service setting, but several patterns distinct from national models emerged. These included heightened risk among younger clients [55] and systematic differences between Urban Honolulu and other counties. These findings point to the ways in which island geography, uneven service distribution, and urban–rural divides create treatment contexts in Hawai’i that would not be adequately captured in national aggregates. Also striking is the discrepancy between TEDS-D reporting and alternative sources such as Hawai’i’s alcohol and drug abuse division data and the N-SUMHSS facility survey. These alternative datasets show that culturally-consonant and community-connected TCs, regarded locally as a ‘gold standard’ for sustained recovery, are substantially underrepresented in federally reported treatment episodes. The likely structural reasons for this underrepresentation (e.g. limited federal or state funding, reporting burdens on nonprofit providers, misalignment between TEDS-D’s episodic model and the long-term continuum of TC care) illustrate how locally-important treatment modalities may be effectively invisible in national analytics. In Hawai’i, where cultural connectedness and community-based recovery models are central, this invisibility is problematic, obscuring the outcomes of approaches that are both clinically effective and culturally appropriate. This limits their limited visibility in policy debates and resource allocation decisions. The Hawai’i-specific analysis thus highlights the need to treat national datasets not as definitive but as partial windows into the treatment landscape, to be interpreted alongside local administrative data and community knowledge.

Future work will focus on methodological, data, and policy advances. Methodologically, future work may deploy quasi-experimental approaches, causal ML, or trajectory modeling of patient EHR to move closer to causal inference and capture longitudinal recovery pathways. Sequence modeling of patient journeys across admission, service setting, LOS, and discharge could reveal patterns obscured in cross-sectional analyses. Data modernization of TEDS-D is also critical: standardized reporting of TCs, mandatory and reason-coded discharge use fields, harmonized length-of-stay bins, and post-discharge follow-up measures would better align national datasets with the lived realities of recovery. Hawai’i in particular illustrates the value of embedding culturally grounded TCs into analytics frameworks, rather than allowing them to remain invisible in national reporting.

Taken together, these findings underscore both the promise and the limitations of explainable ML for national SUD analytics. CatBoost and SHAP offer powerful tools for surfacing interpretable patterns, but their outputs must be situated within the broader structural, cultural, and data-governance landscape. SHAP waterfall and dependence plots enabled interpretability of the model’s predictions, offering a granular view of how features such as employment status, LOS, referral source, and geography shape treatment outcomes. Localized models further improved predictive performance for most states, emphasizing the value of stratified modeling approaches in national health datasets. For policy and operations recommendations, the results point to clear levers: expand access to long-term residential care where appropriate, establish evidence-based minimum lengths of stay, reduce overreliance on detox-only pathways, and integrate employment and housing supports to address stagnancy in SAMHSA’s HHPC domains. The Hawai’i case study provides a concrete demonstration of how local context can sharpen model insight while also revealing reporting blind spots.

## Data Availability

No new data were created or analysed in this study.
